# Formic Acid as an Antimicrobial for Poultry Production: A Review

**DOI:** 10.3389/fvets.2020.00563

**Published:** 2020-09-03

**Authors:** Steven C. Ricke, Dana K. Dittoe, Kurt E. Richardson

**Affiliations:** ^1^Department of Food Science, Center of Food Safety, University of Arkansas, Fayetteville, AR, United States; ^2^Anitox Corporation, Lawrenceville, GA, United States

**Keywords:** formic acid, antimicrobial, food animals, foodborne pathogen, feed, gastrointestinal tract

## Abstract

Organic acids continue to receive considerable attention as feed additives for animal production. Most of the emphasis to date has focused on food safety aspects, particularly on lowering the incidence of foodborne pathogens in poultry and other livestock. Several organic acids are currently either being examined or are already being implemented in commercial settings. Among the several organic acids that have been studied extensively, is formic acid. Formic acid has been added to poultry diets as a means to limit *Salmonella* spp. and other foodborne pathogens both in the feed and potentially in the gastrointestinal tract once consumed. As more becomes known about the efficacy and impact formic acid has on both the host and foodborne pathogens, it is clear that the presence of formic acid can trigger certain pathways in *Salmonella* spp. This response may become more complex when formic acid enters the gastrointestinal tract and interacts not only with *Salmonella* spp. that has colonized the gastrointestinal tract but the indigenous microbial community as well. This review will cover current findings and prospects for further research on the poultry microbiome and feeds treated with formic acid.

## Introduction

Both food animal and poultry production industries are challenged to develop management strategies that achieve a balance between optimizing growth and performance while limiting food safety concerns. Historically, antibiotics fed at subtherapeutic levels were associated with improvements in animal health, welfare, and productivity of animals (1–3). Mechanistically, it has been suggested that antibiotics fed at subinhibitory concentrations mediated their animal host responses via modulation of the gastrointestinal tract (GIT) microbiota and, in turn, their interaction with the host (3). However, continuing concerns over the potential for proliferation of antibiotic-resistant food-associated pathogens and potential association with antibiotic-resistant infections in humans have resulted in the gradual removal of antibiotics for therapeutic use in food animals (4–8). Consequently, the development of feed additives and amendments that meet at least some of these requirements (improvements in animal health, welfare, and productivity of animals) has been an ongoing interest both from an academic research standpoint as well as a commercial development effort (5, 9). Numerous commercial feed additive products have entered into the food animal production market ranging from probiotics and prebiotics to a broad spectrum of essential oils and related compounds from botanical sources as well as chemicals such as aldehydes (10–14). Other commercial feed additives common to the poultry industry are bacteriophages, zinc oxide, exogenous enzymes, competitive exclusion products, and acidic compounds (15, 16).

Among the available choices of chemical feed additives, aldehydes and organic acids have historically been the more extensively studied and utilized group of compounds (12, 17–21). Organic acids, particularly short-chain fatty acids (SCFA), are well-known antagonists to pathogenic bacteria. These organic acids have been employed as feed additives not only to limit the presence of pathogens in feed matrices but also potentially to be active toward general GIT function (17, 20–24). In addition, SCFA result from the fermentation of GIT microbiota harbored in the digestive tract and are believed to play a mechanistic role in the ability of certain probiotics and prebiotics to be antagonistic to pathogens entering the GIT (21, 23, 25).

**Figure 1:**
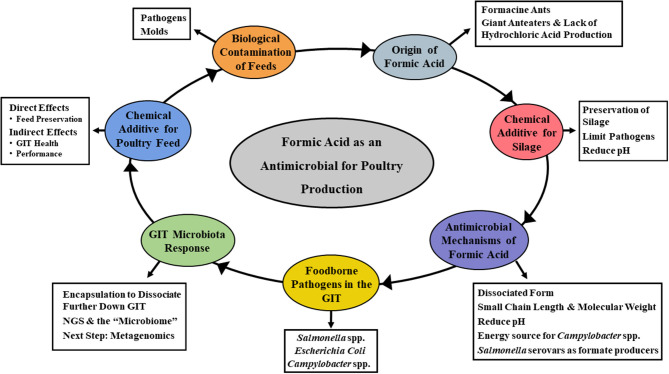
Mind map of the topics covered in the current review. Specifically, focusing on the overall goal of describing the historical and current applications of formic acid as a feed amendment for livestock use, the antimicrobial mechanism(s) attributable to formic acid and how its administration impacts animal and poultry health, and potential approaches for improving efficacy.

Several SCFA have received interest over the years as feed additives. Specifically, propionate, butyrate, and formate have been the subject of numerous research studies and commercial applications (17, 20, 21, 23, 24, 26). While most early interest centered around controlling the occurrence of foodborne pathogens in animal and poultry feeds, the more recent focus has been directed toward animal performance and general promotion of GIT health (20, 21, 24). Acetate, propionate, and butyrate have received considerable attention as organic acid feed additives, with formic acid also being a viable candidate (21, 23). Most of the emphasis to date has focused on food safety aspects of formic acid, particularly on lowering the incidence of foodborne pathogens in livestock feed. However, other aspects of its potential utility are now being considered as well. The overall goal of this review is to discuss the historical and current applications of formic acid as a feed amendment for livestock use (Figure 1). As a part of this, the antimicrobial mechanism(s) attributable to formic acid will be examined. Further elaboration on how this impacts administration in animal and poultry agriculture, and potential approaches for improving efficacy will also be discussed.

## Biological Contamination of Feeds

Food animal and poultry feed production is a complex operation with multiple steps, including physical processing of cereal grains such as grinding to reduce particle size, thermal treatment for pelleting, as well as supplementing the diet with numerous nutritional ingredients depending upon the specific nutrient requirement of the animal (27). Given this complexity, it is not surprising that during feed processing the opportunity to come in contact with numerous environments before the grains reach the feed mill, during feed milling, followed by delivery and feeding of the mixed feed ration occurs (9, 21, 28). Consequently, a highly variable set of microorganisms, including not just bacteria but bacteriophage, fungi, and yeast, have all been identified from feeds over the years (9, 21, 28–31). Some of these contaminants, such as certain fungi, can be problematic for animal health due to their production of mycotoxins (32–35).

Bacterial populations can be relatively diverse and are somewhat dependent on the corresponding methods used for isolation and identification of the microorganisms as well as the source of the samples. For example, microbial compositional profiles might be expected to be somewhat different before thermal processing associated with pelleting (36). While classical culture and plating methodologies have been somewhat informative, more recent applications of next-generation sequencing (NGS) of the microbiome based on the 16S rRNA gene offer a much more comprehensive evaluation of feed microbial communities (9). When Solanki et al. (37) examined the bacterial microbiomes of wheat grains stored over time in the presence of an insect fumigant phosphine, they concluded that the microbiomes were more diverse immediately after harvest and after 3 months of storage. In addition, Solanki et al. (37) demonstrated that Proteobacteria, Firmicutes, Actinobacteria, Bacteroidetes, and Planctomycetes were the dominant phyla among the wheat grains and *Bacillus, Erwinia*, and *Pseudomonas* as being the more predominant genera along with a lesser proportion of *Enterobacteriaceae*. Based on taxonomic comparisons, they concluded that phosphine fumigation altered the bacterial populations considerably but did not influence fungal diversity.

The microbiome-based detection of the genus *Enterobacteriaceae* by Solanki et al. (37) would suggest that feed sources can also harbor foodborne pathogens that could be of public health concern. Foodborne pathogens such as *Clostridium perfringens, Clostridium botulinum, Salmonella* spp., *Campylobacter, Escherichia coli* O157:H7, and *Listeria* have all been associated with animal feeds and silage (9, 31, 38). It is not clear how persistent other foodborne pathogens are in animal and poultry feeds. When Ge et al. (39) sampled over 200 animal feed ingredients, they were able to isolate *Salmonella* spp., generic *Escherichia coli*, and *Enterococcus* but did not detect *E. coli* O157:H7 or *Campylobacter*. However, matrices similar to dry feeds can serve as sources of pathogenic *E. coli*. In tracking the outbreak source of Shiga toxin-producing *E. coli* (STEC) serogroup O121 and O26 associated with human illness occurring in 2016, Crowe et al. (40) used whole-genome sequencing to compare clinical vs. food source isolates. Based on this comparison, they concluded that low moisture raw wheat flour from a flour processing facility was the likely source. The low moisture properties of the wheat flour would suggest that STEC could survive in low moisture animal feeds as well. However, as Crowe et al. (40) pointed out, there were difficulties with isolating STEC from flour samples, and an immunomagnetic-separation approach was required to retrieve sufficient bacterial cells. Similar diagnostic logistics could preclude the detection and isolation of rarely occurring foodborne pathogens in animal feeds as well. Detection difficulties could also be a challenge due to the long term persistence in these types of low moisture matrices. Forghani et al. (41) demonstrated that inoculated mixtures of enterohemorrhagic *E. coli* (EHEC) serogroups O45, O121, and O145 and *Salmonella* (Typhimurium, Agona, Enteritidis, and Anatum) in wheat flour held at room temperature were quantifiable at 84 and 112 days and remained detectable at 24 and 52 weeks, respectively.

Historically, *Campylobacter* species have not been isolated from animal and poultry feeds using conventional culture-based methods (38, 39) even though *Campylobacter* can be readily isolated from the poultry GIT and on poultry meat products (42, 43). However, feed as a potential source may still have some merit. For example, Alves et al. (44) demonstrated that the inoculation of starter and finisher poultry feed with *C. jejuni* followed by storage of the feed at two different temperatures for either 3 or 5 days resulted in the recovery of viable *C. jejuni* and in some cases multiplication. They concluded that *C. jejuni* could undoubtedly survive in poultry feeds and therefore could be a potential source for chickens.

Previously, *Salmonella* spp. contamination of animal and poultry feed has received most of the attention and remains a current focus for the development of detection methods suited explicitly for feeds as well as the pursuit of more effective control measures (12, 26, 30, 45–53). Numerous *Salmonella* spp. isolation and characterization survey studies have been conducted over the years on a wide range of feeds and feed mills (38, 39, 54–61). Collectively, these surveys have revealed that *Salmonella* spp. can be isolated from a diverse set of feed ingredients, feed sources, and types of feeds as well as feed mill operations. Level of prevalence and the predominant *Salmonella* serovar isolates also vary to some extent. For example, Li et al. (57) confirmed the presence of *Salmonella* spp. in 12.5% of the 2,058 total samples collected from complete animal feeds, feed ingredients, pet foods, pet treats, and pet supplements during a collection period from 2002 to 2009. In addition, of the 12.5% confirmed positive *Salmonella* samples, *S*. Senftenberg and *S*. Montevideo were the most prevalent serovars identified (57). In a survey of Texas ready-to-eat and animal feed byproducts, Hsieh et al. (58) reported that fish meal had the highest prevalence of *Salmonella* spp. followed by animal proteins with *S*. Mbanka and *S*. Montevideo being the most frequent serovars identified. Feed mills also represent several potential contamination sites for feeds during mixing and addition of ingredients (9, 56, 61). Magossi et al. (61) were able to demonstrate the potential for multiple contamination sites that occur throughout U.S. feed manufacturing. As a matter of fact, Magossi et al. (61) were able to identify at least one location (of the 12 tested sampling locations) at each of the 11 U.S. feed mills tested across eight states that were culture positive for *Salmonella* spp. Given the potential for *Salmonella* contamination during feed processing, transportation, and daily feeding, it is not surprising that numerous attempts have been made to develop feed additives that decrease microbial contamination and retain these reduced levels throughout the animal production cycle.

## Antimicrobial Mechanisms of Formic Acid

Less is known mechanistically about *Salmonella*'s specific response to formate. Still, Huang et al. (62) noted that formate is present in the mammalian small intestine and that *Salmonella* spp. are capable of producing formate. When Huang et al. (62) examined *Salmonella* virulence gene expression using a series of deletion mutants in critical pathways, they observed that formate could serve as a diffusible signal to induce *Salmonella* invasion of Hep-2 epithelial cells. More recently, Lü et al. (63) isolated a formate transporter, FocA in *Salmonella* Typhimurium, that acts as a specific formate channel at pH 7.0 but also serves as either a passive export channel at high external pH or a secondary active formate/hydrogen ion importer at low pH. However, this work was conducted exclusively on one serovar *S*. Typhimurium. The question remains as to whether all serovars mechanistically respond similarly to formic acid. This question remains a key research question that will need to be addressed in future studies. Regardless of the outcome, it is still prudent to employ multiple *Salmonella* serovars and perhaps even more than one strain for each serovar in screening experiments when general recommendations need to be made for the use of an acid additive to reduce *Salmonella* spp. in feed. Newer approaches such as the ability to genetically barcode strains to distinguish subpopulations of the same serovar (9, 64) offer opportunities to differentiate more subtle differences that could influence variances in conclusions and interpretation.

The chemical and dissociation form of formate may be important as well. In a series of studies, Beier et al. (65–67) demonstrated that inhibition of *Enterococcus faecium, Campylobacter jejuni*, and *Campylobacter coli* correlated with the amount of dissociated formic acid and not pH or undissociated formic acid. The chemical form of formate the bacteria are exposed to appears to matter as well. Kovanda et al. (68) screened several Gram-negative and Gram-positive microorganisms and compared Minimum Inhibitory Concentration (MIC) responses on sodium formate (500–25, 000 mg/L) and a blend of sodium formate and free formate (40/60 w/v; 10–10,000 mg/L). Based on the MIC estimates, they found that sodium formate was only inhibitory to strains of *Campylobacter jejuni, Clostridium perfringens, Streptococcus suis*, and *Streptococcus pneumoniae*, but not *E. coli, Salmonella* Typhimurium, or *Enterococcus faecalis*. Conversely, the blend of sodium formate and free formate was inhibitory to all the microorganisms leading the authors to suggest that free formic acid possesses most of the antimicrobial properties. It would have been interesting to examine different ratios of the two chemical forms to determine whether the range of MIC values correlated with the level of formic acid present in the blended formula vs. responses to 100% formic acid.

Gómez-García et al. (69), have screened essential oils in combination with organic acids such as formic acid against multiple isolates originating from swine, *Escherichia coli, Salmonella* spp. and *Clostridium perfringens*. They tested the efficacy of six organic acids, including formic acid and six essential oils, with formaldehyde as a positive control against the swine isolates. Gómez-García et al. (69) determined the MIC_50_, MBC_50_, and MIC50/MBC_50_ of formic acid to *E. coli* (600 and 2,400 ppm, 4), *Salmonella* spp. (600 and 2,400 ppm, 4) and *Clostridium perfringens* (1,200 and 2,400 ppm, 2), with formic acid performing better out of all the organic acids against *E. coli* and *Salmonella* spp. (69). The explanation for the efficacy of formic acid against *E. coli* and *Salmonella* spp. is its small molecular size and chain length (70).

When Beier and coworkers screened *Campylobacter coli* strains isolated from swine (66) and *Campylobacter jejuni* strains originating from poultry (67), they concluded that the dissociated concentration for formate matched the determined MIC responses as seen with the other organic acids. However, caution was raised as to the relative effectiveness of these acids, including formic acid, since *Campylobacter* is capable of utilizing them as a substrate (66, 67). *Campylobacter jejuni*'s utilization of acids is not surprising as it has been characterized as having a non-glycolytic metabolism. As such, *Campylobacter jejuni* has a limited carbohydrate catabolic capacity and instead relies on gluconeogenesis from amino acids and organic acids for much of its energy metabolism and biosynthesis activities (71, 72). Early work by Line et al. (73) using a phenotype array with 190 carbon sources, noted that a *Campylobacter jejuni* 11168 (GS) could use organic acids as carbon sources, with most being intermediates of the TCA cycle. Further research by Wagley et al. (74) using a carbon utilization phenotype array approach noted that strains of both *Campylobacter jejuni* and *C. coli* examined in their study were able to grow with organic acids as carbon sources. Formic acid specifically serves as a primary energy source of *Campylobacter jejuni* by being a major electron donor for respiratory energy metabolism in *Campylobacter* (71, 75). *C. jejuni* is able to use formic acid as a hydrogen donor via a formate dehydrogenase membrane complex that oxidizes formate to carbon dioxide, protons, and electrons and serves as an electron donor for respiration (72).

## Formic Acid and its Origin in the Insect Class

Formic acid has a long history of being utilized as an antimicrobial feed amendment but also is generated by some insects for use as an antimicrobial defense chemical. Rossini et al. (76) suggested that formic acid was probably the constituent acid in the ant-generated acid juice described nearly 350 years ago by Wray (77). Since then, the understanding of formic acid production by formicine ants and other insects has evolved considerably, and this process is now known to be part of a well-orchestrated toxin defense system for insects (78). Several insect taxa including stingless bees, Oxytrigona (Hymenoptera: Apidae), carabid beetles (*Galerita lecontei* and *G. janus*), stingless formicine ants (subfamily Formicinae), and some moth larvae (Notodontidae, Lepidoptera) are known to produce formic acid as a defense chemical (76, 78–82).

Formicine ants are probably the best characterized and possess an acidophore, a specialized opening that allows them to spray their venom containing formic acid as the primary compound (82). The ant uses serine as a precursor and accumulates large quantities of formic acid in a poison gland that is sufficiently compartmentalized to protect the host ant from the cytotoxic levels of formate until it is dispersed as a spray (78, 83). The emitted formic acid spray can (1) be an alarm pheromone to recruit additional ants, (2) become a defense chemical against competitors and predators, and (3) when combined with tree resin as part of their nest materials, serve as an antifungal and antimicrobial agent (78, 82, 84–88). The antimicrobial properties associated with formic acid production in ants suggests that it could also be applied externally as an additive compound. Brütsch et al. (88) demonstrated this when they added synthetic formic acid to resin resulting in a significantly increased antifungal activity. As further evidence of the potency of formic acid and its biological utility, giant anteaters that lack the ability to produce gastric hydrochloric acid consume ants containing formic acid to provide the concentrated formic acid as a substitute digestive acid (89).

## Formic Acid As A Chemical Additive for Silage

The practical agricultural application of formic acid has been considered and examined for several years. Specifically, formic acid has utility as an additive for animal feed and silage. Both solid and liquid forms of sodium formic acid have been considered safe for all animal species as well as consumers and the environment (90). Based on their assessment (90), a maximum concentration of 10,000 mg formic acid equivalents/kg of feed was deemed safe for all animal species, while 12,000 mg formic acid equivalents/kg of feed were considered safe for swine. Application of formic acid as a feed amendment for animal nutrition has been examined for a number of years. It has been viewed as having commercial value as a preservative in silage and as an antimicrobial for animal and poultry feeds.

Chemical additives such as acids have been an essential element in the management of production and feeding of forage-based silages (91, 92). Borreani et al. (91) noted that achieving optimized, high-quality forage silage production requires stabilizing the forage quality while retaining the maximum amount of dry matter possible. The outcome of this optimization would be minimized losses during all stages of silage from initial aerobic conditions in the silo, followed by fermentation, storage, and reopening the silo for feeding. Specific methods for optimizing silage production in the field and the subsequent silo fermentation have been extensively reviewed elsewhere (91, 93–95) and will not be covered in detail in the current review. A primary concern is yeast- and mold- mediated oxidative deterioration while oxygen remains in the ensiled forage (91, 92). Consequently, biological inoculants, and chemical additives were introduced to counter the detrimental impact of deterioration (91, 92). Additional concerns for silage additives include limiting the proliferation of pathogens such as pathogenic *E. coli, Listeria*, and *Salmonella* spp. that may be present in the silage as well as mycotoxin producing fungi (96–98).

Muck et al. (92) have categorized acid additives in two distinct groups. Acids such as propionic, acetic, sorbic, and benzoic acids retain aerobic stability of silage while being fed to ruminants by limiting yeasts and molds (92). Muck et al. (92) delineated formic acid from the other acids as a direct acidifier that can suppress clostridia and spoilage microorganisms while preserving silage protein integrity. For the practical application of the acids, their corresponding salt form represents the more common chemical version employed to avoid corrosiveness of the non-salt versions of these acids (91). Formic acid has also been investigated as an acid additive for silage by numerous research groups. It is known for its rapid acidification potential and inhibitory action on the growth of undesirable silage microorganisms that reduce levels of silage forage protein and water-soluble carbohydrates (99). As such, He et al. (100) demonstrated the ability of formic acid to suppress coliforms and decrease the pH of the silage. Formic acid and cultures of lactic acid-producing bacteria have also been added to silage to promote acidification and organic acid production (101). In fact, Kuley et al. (101) determined that lactic and formic acid were produced in amounts exceeding 800 and 1,000 mg organic acid/100 g sample when silage was acidified with 3% (w/v) of formic acid. Muck et al. (92) have extensively reviewed the silage additive research literature, including studies focused on and/or including formic and other acids that were published since the year 2000. Therefore, these individual research studies will not be discussed in detail in the current review except to summarize a few key points regarding formic acid efficacy as a silage chemical additive. Both non-buffered and buffered formic acid have been examined, and in most cases clostridial spp. and their associated activities (consumption of carbohydrates, proteins and lactic acid, and the excretion of butyric acid) tended to decline along with decreases in ammonia and butyrate production and improved retention of dry matter (92). There were some limits to the impact of formic acid, but combinations with other acids as silage additive blends appeared to overcome some of these issues (92).

Formic acid may limit pathogenic organisms linked to human public health concerns. For example, Pauly and Tham (102) inoculated *Listeria monocytogenes* into small laboratory silos containing ryegrass at three different dry matter levels (200, 430 and 540 g/kg), followed by incorporating either formic acid (3 mL/kg) or lactic acid bacteria (8 × 10^5^/g) with cellulolytic enzymes. They reported that either treatment reduced *L. monocytogenes* to non-detectable levels in the low dry matter silage (200 g/kg). However, in the medium-dry matter silage (430 g/kg), *L. monocytogenes* could still be quantified at 30 days in formic acid treated silage. The reduction in *L. monocytogenes* appeared to correspond to a lower pH, levels of lactic acid, and pooled undissociated acids. Therefore, Pauly and Tham (102) alluded to the fact that levels of lactic acid and pooled undissociated acids were especially important and were probably the reason why the reduction in *L. monocytogenes* was not observed in the formic acid treated medium in the higher dry matter silage. In the future, similar studies will need to be conducted with other common silage pathogens such as *Salmonella* spp. and pathogenic *E. coli*. A more comprehensive 16S rDNA sequence profiling of the entire silage microbial community could also help identify overall silage microbial population shifts occurring during the various stages of silage fermentation in the presence of formic acid (103). Generating microbiome data may provide analytical support to better predict the progress of silage fermentation as well as design optimal additive combinations to maintain high-quality forage silage.

## Formic Acid and Antimicrobial Activities in Animal Feeds

For cereal grain-based animal diets, formic acid has been employed as a feed antimicrobial to limit pathogen levels in a wide range of feed matrices originating from cereal grains as well as specific feed ingredients such as animal byproducts. Impact on pathogen populations in poultry and other animals can be broadly categorized as either direct effects on pathogen populations in the feed itself or the more indirect effect on pathogens colonizing the animal's GIT after the treated feed has been consumed (20, 21, 104). Obviously, these two categories are interconnected as a reduction of pathogens in the feed should lead to less colonization when the feed is consumed by the animal. However, several factors can potentially influence the antimicrobial properties of the particular acid introduced to a feed matrix such as feed composition, and form of the acid administered (21, 105).

Historically, much of the focus for the application of formic acid and other related acids has been on the direct control of *Salmonella* spp. in animal and poultry feeds (21). The results of these studies have been summarized in details in several reviews that have been published at different times (18, 21, 26, 47, 104–106) and therefore, only some of the key conclusions from these studies will be discussed in the current review. Several studies have indicated that the antimicrobial activity of formic acid in the feed matrix is dependent on the dose and exposure time of formic acid, the moisture content of the feed matrix, and the bacterial concentration of the feed and animal GIT (19, 21, 107–109). The type of feed matrix and the origin of animal feed ingredients are also factors. Consequently, several studies have indicated that level of *Salmonella* spp. recovered from animal byproducts may differ compared to their plant-based counterparts (39, 45, 58, 59, 110–112). However, some of these differences in response to acids, such as formate, may be related to serovar survival differences in feed and temperature of feed treatment (19, 113, 114). Serovar differences in response to acid treatment may also be a factor in poultry infection by contaminated feed (113, 115) and differences in virulence gene expression (116) could play a role. Differences in acid tolerance could in turn influence detection of *Salmonella* spp. on culture media if the acid that carries over from the feed is not adequately buffered (21, 105, 117–122). The physical form of the diet in terms of particle size may also contribute to the relative effectiveness of formic acid in the GIT (123).

Strategies to optimize the antimicrobial activity of formic acid addition to feed also appears to be critical. Application of acids at higher concentrations in feed ingredients that are at a high-risk of contamination prior to feed mixing has been suggested to minimize potential feed mill equipment damage and animal palatability issues (105). Jones (51) concluded that *Salmonella* spp. present in the feed before chemical decontamination might be more challenging to limit than those that come in contact with the feed after chemical treatment. Thermal treatment of feeds during feed mill processing is considered an intervention for limiting *Salmonella* spp. contamination in feeds but depends on feed composition, particle size, among other factors associated with the milling process (51). The antimicrobial activity of acids is also impacted by temperature, and increased temperature in the presence of organic acids can elicit a synergistic inhibition of *Salmonella* spp. as observed in liquid cultures of *Salmonella* (124, 125). Several studies on *Salmonella* spp. contaminated feed have supported the idea that increased temperature improved the efficacy of the acids incorporated in the feed matrix (106, 113, 126). Using a central composite design, Amado et al. (127) examined the interaction between temperature and acids (formic or lactic acid) on 10 *Salmonella enterica*, and *E. coli* isolates from various cattle feeds and inoculated into acidified pelleted cattle feed. They concluded that heat was the more dominant influential factor on microbial reduction with the type of acid and bacterial isolate also being a factor. Synergism with the acids still generally occurred, allowing for the potential to use lower temperatures and lower acid concentrations. However, they also noted that synergy did not always occur with formic acid, leading them to suspect that either volatilization of formic acid occurred at higher temperatures or buffering by feed matrix components was a factor.

## Impact on Foodborne Pathogens in the Gastrointestinal Tract

Limiting foodborne pathogens in the feed during storage prior to feeding animals is undoubtedly a means to control their introduction to the animal during consumption of the feed. However, acids in the feed have the opportunity as they enter into the GIT to continue to exhibit antimicrobial activities. Externally introduced acid antimicrobial activity in the GIT is potentially dependent on numerous factors including GIT acid concentration, GIT site of activity, level of GIT pH and oxygen, age of the animal, and the corresponding composition of microbial populations inhabiting the GIT as a function of GIT location and animal maturity (21, 24, 128–132). In addition, the resident GIT anaerobic microbial population, which becomes more dominant in the lower GIT sections of the monogastric animal as it matures, is actively producing organic acids via fermentation, which, in turn, are also potentially antagonistic to transient pathogens entering the GIT (17, 19–21).

Most of the early work focused on using organic acids, including formate, to limit *Salmonella* spp. in the poultry GIT, which has been discussed in detail in several reviews (12, 20, 21). From an overview of these studies, a few key observations have prevailed. McHan and Shotts (133) reported that feeding formic and propionic acid reduced cecal levels of *S*. Typhimurium inoculated in young chicks and quantified at 7, 14, and 21 days of age. However, when Hume et al. (128) tracked C^−14^ labeled propionate, they concluded that very little propionate in the feed likely reached the ceca. Whether this is true of formic acid remains to be determined. However, more recently, Bourassa et al. (132) did note that feeding formic acid at 4 g per ton for a 6 week grow-out period in broiler chicks reduced cecal *S*. Typhimurium concentrations below detection levels.

The presence of formic acid in the diet likely influences other poultry GIT compartments. Al-Tarazi and Alshawabkeh (134) demonstrated that a formic and propionic acid mixture decreased the frequency of *S*. Pullorum in both the crop and the ceca. Thompson and Hinton (129) observed that a commercial blend of formic and propionic acid resulted in an increased concentration of these two acids in the crop and gizzard and, when representative crop conditions were simulated *in vitro*, were bactericidal to *S*. Enteritidis PT4. This is supported by *in vivo* data when Byrd et al. (135) added formic acid to the drinking water of broilers undergoing a simulated pre-transport feed withdrawal similar to that experienced by broilers prior to transit to the poultry processing plant. The presence of formic acid in the drinking water resulted in reduced *S*. Typhimurium crop and cecal populations along with a decrease in the frequency of *S*. Typhimurium positive crops, but not the number of positive ceca (135). Developing delivery systems that serve to protect organic acids as they enter the GIT to remain active in the lower compartments may help to increase efficacy. For example, protecting formic acid by microencapsulation for administration in feed has been shown to decrease *S*. Enteritidis in cecal contents (136). However, this may differ among animal species. For example, Walia et al. (137) did not see *Salmonella* spp. reduction in 28-day old pigs fed an encapsulated blend of formic acid, citric acid, and essential oils in either the cecal digesta or lymph nodes although *Salmonella* spp. shedding in the feces was reduced on day 14 but not on day 28. They did suggest that the horizontal transfer of *Salmonella* spp. among pigs was prevented.

While the majority of the research on formic acid as an antimicrobial in food animal production has focused on foodborne *Salmonella* spp., there have been some studies with other pathogens inhabiting the GIT. As indicated by the *in vitro* work of Kovanda et al. (68), formic acid may be effective against other GIT foodborne pathogens as well, including *E. coli* and *Campylobacter jejuni*. Early research indicated that organic acids, such as lactic acid and commercial blends that contained formic acids as one of several components, could lower *Campylobacter* levels in poultry (135, 138). However, employing formic as an antimicrobial agent against *Campylobacter* may need some caution exercised, as noted earlier by Beier et al. (67). This fact may be particularly problematic for poultry diet supplementation since formic acid serves as a major energy donor for *Campylobacter jejuni* respiration. In addition, it is believed that part of its ecological niche in the GIT is to metabolically cross-feed on the mixed acid fermentation products such as formic acid produced by GIT bacteria (139). There is some support for this. Because formic acid is a chemoattractant to *Campylobacter jejuni*, double mutants impaired in both formate dehydrogenase and hydrogenase display decreased cecal colonization in broilers compared to the wild-type *Campylobacter jejuni* strain (140, 141). It is not known how much external formic acid supplementation could influence *Campylobacter jejuni* establishment in the chicken GIT. Several variables could impact this as the actual GIT formic acid concentration could be lower due to catabolism of formic acid by other GIT bacteria or absorption of formic acid in the upper part of the GIT. Also, formic acid is a potential fermentation product generated by some GIT bacteria, and this could contribute to overall formic acid GIT levels. Quantitation of formic acid in GIT contents and metagenomics to identify formate dehydrogenase genes would potentially provide some clarity of formic acid microbial ecology.

Roth et al. (142) compared broilers fed either the antibiotic enrofloxacin or an acid blend of formic acid, acetic acid, and propionic acid on the prevalence of antibiotic-resistant *E. coli*. Total *E. coli* and antibiotic-resistant *E. coli* isolates were enumerated from pooled fecal samples of 1-day-old broiler chicks and cecal contents of 14- and 38-day-old broilers. *E. coli* isolates were screened for resistance to ampicillin, cefotaxime, ciprofloxacin, streptomycin, sulfamethoxazole, and tetracycline based on the breakpoint concentration for each respective antibiotic as previously defined. When the respective *E. coli* populations were quantified and characterized, neither the enrofloxacin nor the acid blend supplementation altered the total *E. coli* recovered from 17 and 28-day old broiler ceca. Birds receiving diets supplemented with enrofloxacin yielded increased levels of ciprofloxacin, streptomycin, sulfamethoxazole, and tetracycline-resistant *E*. coli in the ceca, but a decrease in cefotaxime resistant *E. coli*. The blended acids resulted in decreased numbers of ampicillin- and tetracycline-resistant cecal *E. coli* compared with both control and enrofloxacin-supplemented birds. The blended acids also resulted in fewer ciprofloxacin- and sulfamethoxazole-resistant *E*. coli in the ceca vs. the enrofloxacin supplemented birds. It is not clear mechanistically how acids could reduce antibiotic-resistant *E. coli* without reducing the total numbers of *E. coli*. However, the outcome of the study performed by Roth et al. (142) may be evidence for the reduction of dissemination of antibiotic-resistant genes among *E. coli*, such as the plasmid conjugation inhibitors described by Cabezón et al. (143). It would be interesting to conduct a more in-depth profile of plasmid-mediated antibiotic resistance in poultry GIT populations in the presence of feed additives such as formic acid and further develop this profile with an assessment of the GIT resistome.

## Interaction of the Non-Pathogen Gastrointestinal Microbiota With Formic Acid

Developing optimal antimicrobial feed additives while targeting pathogens ideally should have minimal impact on the overall GIT microbiota, particularly microbial members that would be considered beneficial to the host. However, a deleterious impact on the resident GIT microbial population can occur in the presence of externally introduced organic acids and could, to some extent, offset their pathogen prevention benefits. For example, Thompson and Hinton (129) observed decreases in layer hen crop lactic acid in birds fed a formic acid-propionic acid blend suggesting that the presence of these external organic acids in the crop caused a decrease in the crop lactic acid bacterial population. The presence of lactic acid bacteria in the crop is considered a barrier to *Salmonella* spp., so disrupting this resident crop microbiota could be problematic for achieving a successful reduction in *Salmonella* GIT colonization (144). Less impact may occur in the lower part of the avian GIT as Açikgöz et al. (145) did not detect differences in total intestinal bacteria or *E. coli* in 42-day-old broilers receiving formic acid acidified water. As the authors speculated, this might be due to the formic acid being metabolized in the upper part of the GIT as noted by others for externally introduced SCFA (128, 129).

### The Case for Microencapsulation

Protection of formic acid via some form of encapsulation might offer a means to reach lower sections of the GIT. Willamil et al. (146) observed that microencapsulating formic acid significantly increased total SCFA in the ceca of pigs compared to those fed non-protected formic acid. This outcome led the authors to suggest that formic acid, if sufficiently protected, can effectively reach the lower GIT compartments. However, several other measurements, such as formic acid and lactate concentration, although higher than control diet-fed pigs, were not statistically different from non-protected formic acid-fed pigs. *Lactobacilli* populations were not changed by any of the treatments even though lactic acid was increased nearly three-fold in pigs fed either both unprotected or protected formic acid. It may be possible that differences would be more distinct with other lactic acid-producing cecal microorganisms (1) that were not detected with these methods and/or (2) whose metabolic activities were impacted to change fermentation patterns such that more lactic acid was being produced by the resident lactic acid bacterial population.

### Enhanced Resolution—The Impact of Formic Acid on Poultry GIT Microbiota

To better delineate feed additive impact on the food animal GIT, microbiological identification methodologies with increased resolution are required. In the past few years, NGS of the 16S RNA gene for microbiome taxonomic identification and microbial community diversity comparisons (147) have made it possible to develop a better understanding of the interactions between dietary feed additives and the GIT microbiota of food animals such as poultry.

A few studies have incorporated microbiome sequencing assessment of the chicken GIT microbial consortia response to formic acid supplementation. Oakley et al. (148) conducted a study with 42-day-old broilers fed different combinations of formic, propionic, and medium-chain fatty acids administered either in the drinking water or feed. Seeder birds were inoculated with nalidixic acid-resistant *Salmonella* Typhimurium, and ceca were removed at 0, 7, 21, and 42 days of age. Cecal samples were prepared for 454 pyrosequencing and the sequence results assessed for taxonomic classification and similarity comparisons. In general, treatments had little impact on the cecal microbiome or levels of *S*. Typhimurium. However, in general, levels of recovered *Salmonella* spp. decline as the birds become older, and this was supported by the taxonomic microbiome analyses where the relative abundance of *Salmonella* sequences also declined over time. The authors noted that the most significant shifts in GIT microbiota occurred over time across all treatments as cecal microbial populations became more diverse over time as the broilers matured. In a more recent study, Hu et al. (149) compared drinking water and feed delivery of an organic acid blend (formic, acetic, and propionic acids and ammonium formate) with a Virginiamycin supplemented diet on broiler cecal microbiomes from samples collected during two phases (1–21 days and 22–42 days). While some cecal microbiome diversity differences among treatment were detectable in birds at 21 days, by the time birds reached 42 days of age, no differences in alpha or beta diversity were detected. The lack of differences at 42 days of age led the authors to suggest that growth performance benefits may be linked to the earlier establishment of an optimally diversified microbiota.

Microbiome analyses exclusively focused on the cecal microbial populations may not be reflective of where most of the dietary organic acid influence is occurring in the GIT. The upper GIT microbiome populations of broilers may be more likely impacted by dietary organic acids, as indicated by the results from Hume et al. (128). Hume et al. (128) demonstrated that most of the externally supplemented propionate is absorbed in the avian upper GIT. There are also more recent GIT microbial characterization studies that support this. Nava et al. (150) demonstrated that the combination of an organic acid blend [^DL^-2-hydroxy-4(methylthio) butanoic acid], formic, and propionic acid (HFP) impacted the intestinal microbial populations and increased the *Lactobacillus* spp. colonization of the chick ileum. More recently, Goodarzi Boroojeni et al. (151) examined two levels (0.75 and 1.50%) of a formic and propionic acid blend fed to broiler chicks for 35 days. At the termination of the experiment, the crop, gizzard, distal two-thirds of the ileum, and ceca were removed and sampled for RT-PCR quantitation of specific GIT bacterial groups and GIT metabolites. In the crop, neither concentration of organic acids altered the *Lactobacillus* spp. or *Bifidobacterium* spp. populations, but did increase the *Clostridial* clusters. In the ileum, the only changes that occurred were decreases in *Lactobacillus* spp. and *Enterobacteria* vs. no changes in any of these bacterial groups in the cecum (151). Total lactate (D and L) concentrations were reduced for the highest level of organic acid additive in the crop, and both organic acid levels in the gizzard, the lower organic acid concentration in the cecum. No shifts occurred in the ileum. As for SCFA, only propionate was altered in the crops and gizzards of birds receiving organic acids. There was nearly a ten-fold increase of propionate in the crops of birds receiving the lower organic acid concentration and an eight- and fifteen-fold increase in the gizzard for the two levels of organic acids. There was less than a two-fold increase in acetic acid in the ileum. Collectively these data support the idea that most of the external organic acid additive influence occurs in the crop with minimal impact of organic acids on the lower GIT microbial populations and suggests that fermentation patterns may be altered in the resident populations of the upper GIT.

Clearly, more microbiome characterization is warranted to achieve sufficient elucidation of microbial responses to formic acid throughout the GIT. More emphasis on in-depth analyses of specific GIT compartmental microbial taxonomy, particularly in the upper GIT sections such as the crop, could offer more explanations for understanding the selection of particular groups of microorganisms. Their metabolic and fermentation activities could also establish whether their relationship to pathogens entering the GIT would be antagonistic. It would also be of interest to conduct metagenomic analyses to see if more “acid-tolerant” resident bacteria are selected with exposure to acidic chemical additives that are fed to the birds over their lifetime and if either the presence and/or metabolic activity of these bacteria create additional barriers to pathogen colonization.

## Conclusions

Formic acid has been used as a chemical animal feed additive and silage acidifier for several years. One of its main applications has been as an antimicrobial to limit pathogens in the feed and their subsequent establishment in the avian GIT. Formic acid has been shown to be a relatively effective antimicrobial against *Salmonella* spp. and other pathogens based on *in vitro* model studies. Still, it may be more limited in feed matrices due to the high organic matter and potential buffering capacity of the feed components. Once consumed with feed or through the drinking water, formic acid appears to be antagonistic to *Salmonella* spp. and other pathogens. Still, most of this occurs in the upper compartments of the GIT as the formic acid concentration probably diminishes in the lower GIT, as is known to occur for propionate. The concept of protection of formic acid via encapsulation offers a potential means for the delivery of more acid to the lower GIT. In addition, blends of multiple organic acids have been suggested as being more efficacious at enhancing bird performance rather than the administration of single acids (152). *Campylobacter* in the GIT may differ in its response to formic acid since it can use it as an electron donor, and thus the acid serves as a primary energy source. It has not been established whether increasing GIT formic acid concentration would favor *Campylobacter*, and this still may not occur depending on other GIT organisms that may be capable of using formic acid as a substrate.

More research needs to be conducted on the impact of GIT formic acid on non-pathogenic indigenous GIT microorganisms. Selective antagonism of pathogens without disruption of the members of the GIT microbial community considered beneficial to the host would be preferred. However, this requires more in-depth microbiome sequence analyses of these resident GIT microbial consortia. While some research has been reported on the cecal microbiome in birds fed formic acid, more emphasis needs to be placed on the upper GIT microbial communities. Identification of microorganisms and comparison of similarities among GIT microbial groups in the presence or absence of formic acid may not be the complete narrative. Other analyses, including metabolomics and metagenomics, are also needed to characterize the functional differences among compositionally similar populations. This characterization will be necessary for establishing linkages between the GIT microbial population and bird performance responses to the formic acid amendment. Combining methods to better define GIT function should lead to more effective organic acid supplementation strategies and, ultimately, better predictability for optimal bird health and performance while limiting food safety risks.

## Author Contributions

SR wrote the review with assistance from DD and KR. All authors significantly contributed to the work of the current review.

## Conflict of Interest

KR works for Anitox Corporation. The remaining authors declare that the research was conducted in the absence of any commercial or financial relationships that could be construed as a potential conflict of interest.
